# The Posterior Tibial Slope Is Not Associated With Graft Failure and Functional Outcomes After Anatomic Primary Isolated Anterior Cruciate Ligament Reconstruction

**DOI:** 10.1177/03635465231209310

**Published:** 2023-11-17

**Authors:** Maximilian Hinz, Moritz Brunner, Philipp W. Winkler, José Fernando Sanchez Carbonel, Lorenz Fritsch, Romed P. Vieider, Sebastian Siebenlist, Julian Mehl

**Affiliations:** † Department of Sports Orthopaedics, Technical University of Munich, Munich, Germany; ‡ Department of Orthopaedics and Traumatology, Kepler University Hospital, Linz, Austria; Investigation performed at the Department of Sports Orthopaedics, Technical University of Munich, Munich, Germany

**Keywords:** ACL, return to sport, patient-reported outcome, revision, reinjury, risk factor

## Abstract

**Background:**

Biomechanical studies have shown that an increased medial posterior tibial slope (MPTS) may affect anteroposterior knee laxity and tibial shear forces, ultimately increasing the risk for graft failure after anterior cruciate ligament (ACL) reconstruction. Previous clinical studies have, however, reported inconclusive results.

**Purpose:**

The purpose of this study was to evaluate the relationship between the MPTS and graft failure as well as functional outcomes after anatomic primary isolated ACL reconstruction using a hamstring tendon autograft. It was hypothesized that an increased MPTS would be associated with a higher ACL graft failure rate. Furthermore, a higher MPTS would negatively correlate with functional outcomes in patients without ACL graft failure.

**Study Design:**

Case-control study; Level of evidence, 3.

**Methods:**

Consecutive patients who underwent isolated primary ACL reconstruction with an anteromedial portal drilling technique between January 2011 and December 2019 were retrospectively reviewed. The MPTS was measured on preoperative lateral knee radiographs. At a minimum of 24 months postoperatively, the ACL graft failure rate and patient-reported outcome measures (PROM; International Knee Documentation Committee subjective knee form, Lysholm score, Tegner Activity Scale, visual analog scale for pain and subjective instability) were evaluated. Differences in the MPTS between patients with and without ACL graft failure as well as the frequency of graft failure between those with an MPTS <12° and those with an MPTS ≥12° were assessed for statistical significance. Binary logistic regression analysis was performed to stratify the risk of graft failure with the following variables: MPTS, age at surgery, and sex. Correlation analysis was performed to evaluate the relationship between the MPTS and PROM in patients without ACL graft failure.

**Results:**

In total, 326 patients were included (median follow-up, 71.0 months [IQR, 49.0-104.0 months]). There was no significant difference in the MPTS between patients with and without graft failure (10.6°± 3.2° vs 11.2°± 2.8°, respectively; *P* = .264). Additionally, there was no significant difference in the frequency of graft failure between patients with an MPTS <12° and those with an MPTS ≥12° (15.6% vs 16.5%, respectively; *P* = .835). Binary logistic regression showed that younger age at the time of surgery (odds ratio, 1.069 [95% CI, 1.031-1.109]) was associated with graft failure; sex and MPTS were not associated with graft failure. In patients without ACL graft failure, there was no significant correlation between the MPTS and PROM.

**Conclusion:**

In patients who underwent anatomic primary isolated ACL reconstruction, an increased MPTS was not associated with a higher rate of graft failure or inferior functional outcomes. Younger age was a significant nonmodifiable risk factor for ACL graft failure.

The significance of the posterior tibial slope (PTS) in the context of anterior cruciate ligament (ACL) injuries has been discussed controversially in the current literature. It has been shown that a steeper PTS leads to increased anterior tibial shear forces and anterior tibial translation,^1,13,30^ which may increase the risk for ACL injuries in native^13,24,25,30,36^ and ACL-reconstructed knees.^1,21^ Giffin et al,^13^ conversely, found no association between the PTS and in situ forces in the ACL. Although some clinical studies found that a steeper PTS, frequently reported as ≥12°, increased the risk for primary ACL injuries or injury recurrence after ACL reconstruction,^§^ the findings of other studies are in disagreement.^5,7,19,33^

The influence of the PTS on graft failure and functional outcomes in patients undergoing primary isolated ACL reconstruction with an anteromedial portal drilling technique has been insufficiently investigated thus far. It is crucial to assess the influence of the PTS on ACL graft failure in patients without concomitant injuries that require surgical treatment, as they have a confounding effect on outcomes after ACL reconstruction.^6^ Therefore, the aim of this study was to evaluate the effect of an increased PTS on ACL graft failure and, in patients without ACL graft failure, functional outcomes. It was hypothesized that a steeper PTS would be associated with ACL graft failure and, in patients without ACL graft failure, inferior functional outcomes.

## Methods

Patients who underwent primary isolated single-bundle ACL reconstruction with an anteromedial portal drilling technique using a hamstring tendon autograft at the authors’ institution between January 2011 and December 2019, with a minimum follow-up of 24 months postoperatively, were eligible to participate. Patients with a minimum age of 16 years at the time of surgery were included. Exclusion criteria were previous ipsilateral knee surgery, concomitant abnormalities that needed to be treated at index surgery, and malrotated or short (tibial shaft length <15 cm) preoperative lateral knee radiographs. ACL graft failure was defined as complete ACL graft disruption, confirmed by magnetic resonance imaging (MRI), or revision ACL reconstruction.

The present study was approved by the ethics committee of the Technical University of Munich (reference No. 9/22-S-NP) and conducted according to the Declaration of Helsinki. All patients signed written informed consent forms.

### Data Collection of Patient and Surgical Information

Patient-specific data included age at the time of primary ACL reconstruction, sex, laterality of the injury, and body mass index. Additionally, injury- and surgery-related data were collected and analyzed: time from the injury to surgery (weeks), and graft diameter (in millimeters).

### Patient-Reported Outcome Measures

At follow-up, patient-reported outcome measures (PROM) including the Lysholm score, International Knee Documentation Committee subjective knee form, Tegner Activity Scale, visual analog scale for pain, and subjective instability (0-10 scale) were administered.

### Radiographical Evaluation

**Figure 1.**
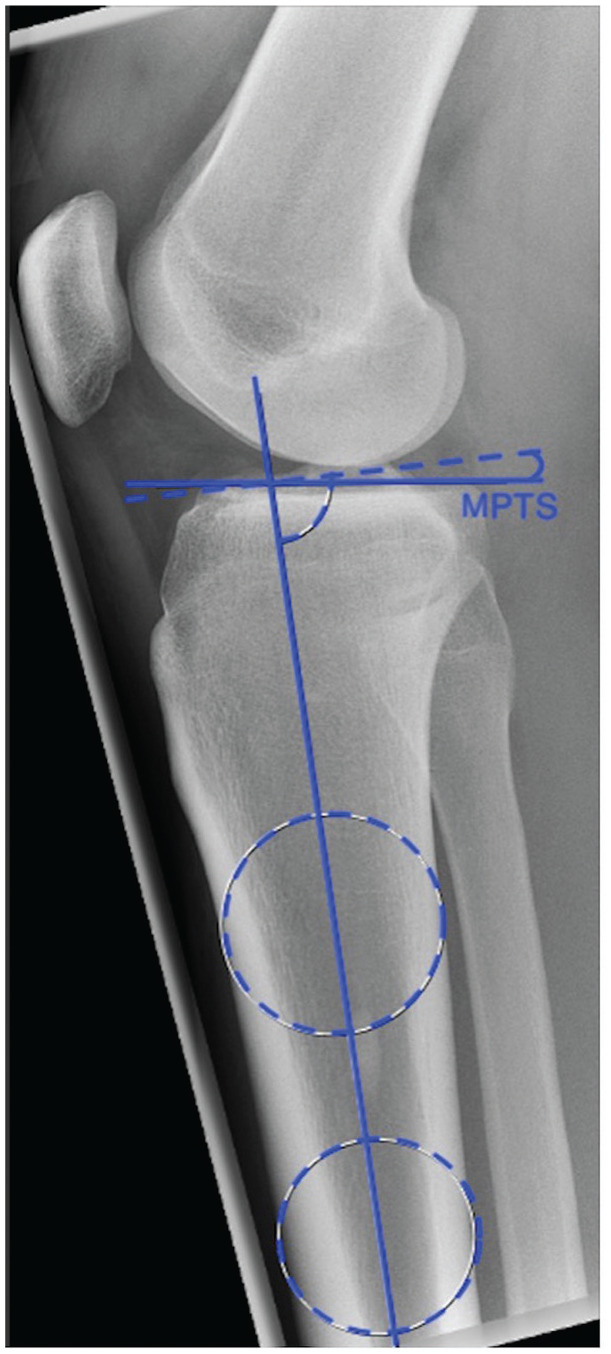
Measurement of the medial posterior tibial slope (MPTS). The MPTS was defined as the angle between the tibial shaft axis and a tangential line along the medial tibial plateau, subtracted from 90°.

The medial PTS (MPTS) was measured on lateral knee radiographs with a minimum tibial shaft length of 15 cm and a posterior femoral condylar distance ≤6 mm^35,39^ according to the method described by Dejour and Bonnin.^8^ In brief, the MPTS was determined as the angle between the proximal tibial shaft axis and a tangential line to the medial tibial plateau, subtracted from 90° of the diaphyseal axis. The tibial shaft axis was defined as a line connecting 2 midpoints, one 5 cm below the tibial tuberosity and the second 15 cm distal to the tibial joint line, between the anterior and posterior tibial cortices (Figure 1). All measurements were performed by an independent observer (M.B.) on the best available lateral knee radiographs of the patients’ injured knee using an image analysis system (iSite PACS; Philips), which had a measurement accuracy of 0.1 mm and 0.1°. The observer was not involved in the treatment of the patients and was blinded to whether the patients reported ACL graft failure.

### Surgical Technique

All patients underwent anatomic single-bundle ACL reconstruction using an autologous hamstring tendon graft (semitendinosus tendon alone or combined with gracilis tendon). The femoral tunnel was drilled via an anteromedial portal, and the tibial tunnel was prepared in the center of the ACL tibial footprint according to the graft size. Graft fixation was performed in 30° of flexion using an extracortical suspension device (ACL TightRope; Arthrex) on the femoral side and a biocomposite interference screw (BioComposite FastThread; Arthrex) on the tibial side.

### Postoperative Rehabilitation

Partial weightbearing (20 kg) was allowed for the first 2 weeks postoperatively. For the first 6 weeks, a knee brace was recommended (M.4s; medi). Physical therapy started on the first postoperative day with passive flexion, with patients attending sessions 2 to 3 times per week. Return to activities, such as running and cycling, was encouraged from the 12th postoperative week. Return to pivoting sports was recommended ≥12 months postoperatively.

### Statistical Analysis

An a priori sample size calculation was conducted based on a previous study by Webb et al^38^ in which it was shown that an increased MPTS is associated with an increased risk of further ACL injuries. Therefore, a mean MPTS of 9.6°± 2.3° for patients with ACL graft failure and a mean MPTS of 8.5°± 2.3° for patients without ACL graft failure were assumed. Moreover, a group allocation of 31 for ACL graft failure and 131 for no ACL graft failure was chosen.^38^ Based on these data, a total sample size of 222 patients (ACL graft failure: n = 43; no ACL graft failure: n = 179) was necessary to achieve a statistical power of 0.8 (effect size = 0.48; alpha = .05). Power analysis was conducted using G*Power (Heinrich Heine University Düsseldorf).^12^

Data were analyzed using SPSS (Version 28.0; IBM). Categorical variables are presented as counts and percentages. The normal distribution of continuous variables was assessed by the Shapiro-Wilk test. Normally distributed continuous variables are reported as the mean ± SD. Nonnormally distributed continuous variables are reported as the median (interquartile range). For group comparisons of continuous variables, an unpaired *t* test was applied, and for group comparisons of ordinal variables, the Mann-Whitney *U* test was applied. For group comparisons of categorical variables, the chi-square test or the Fisher exact test was applied, as appropriate. Binary logistic regression analysis was performed to assess the relative contribution of age, sex, and MPTS on the occurrence of ACL graft failure. Overall, 3 factors were selected to ensure the statistical power of regression analysis. Age and sex were selected because of their association with ACL graft failure in previous statistical analyses and thus were further explored as possible factors in regression analysis. The MPTS represented the core of this study and was therefore also selected as a factor. Likewise, recent systematic reviews^43^ and previous studies^7,16,22,38^ have indicated that these factors may have an effect on graft failure. Spearman rank correlation coefficients were used to evaluate the association between the MPTS and PROM (International Knee Documentation Committee subjective knee form, Lysholm score, Tegner Activity Scale, visual analog scale for pain, and subjective instability). Statistical significance was set at a *P* value of <.05.

A subset of 30 lateral knee radiographs was randomly selected and assessed by 2 authors (M.H. and M.B.) to evaluate intraclass correlation coefficients and, subsequently, calculate the intrarater and interrater reliability. Both the intrarater and interrater reliability were “almost perfect” for MPTS measurements (intrarater reliability: 0.852; interrater reliability: 0.915).

## Results

**Figure 2.**
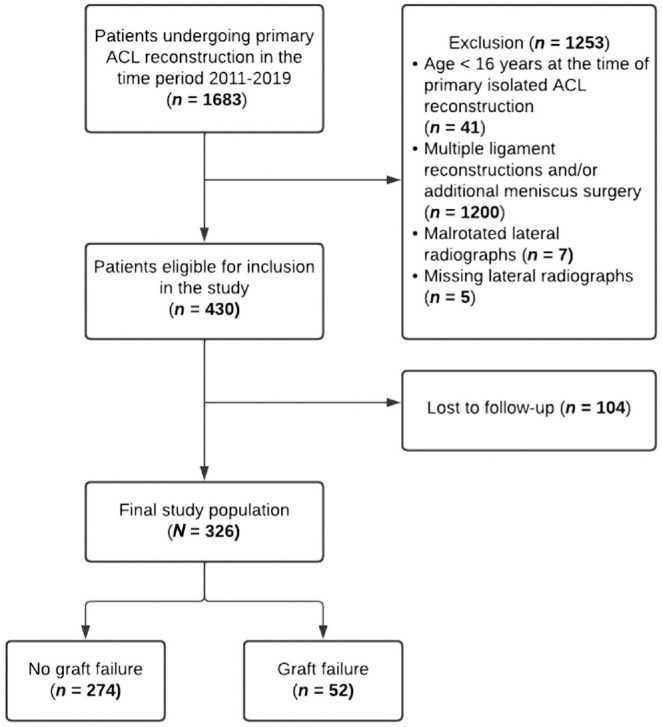
Flowchart of patient enrollment. ACL, anterior cruciate ligament.

In total, 326 patients were included at a median follow-up of 71.0 months (IQR, 49.0-104.0 months). Details on patient characteristics and patient enrollment are given in Table 1 and Figure 2, respectively.

**Table 1:** Characteristics of Study Cohort^*a*^

	Value (n = 326)
Age at primary reconstruction, y	29.0 (21.0-37.3)
Male sex, n (%)	168 (51.5)
Body mass index	23.7 (21.6-25.6)
Follow-up, mo	71.0 (49.0-104.0)
Right laterality of injury, n (%)	153 (46.9)
Time from injury to surgery, mo	8.4 (5.4-15.6)
Graft diameter, mm	8.0 (7.5-8.5)

a Data are expressed as median (interquartile range) unless otherwise specified.

### Graft Failure

Graft failure was reported in 52 patients (15.9%). There was no significant difference regarding the MPTS in patients with versus without ACL graft failure (10.6°± 3.2° vs 11.2°± 2.8°, respectively; *P* = .264). There was no significant difference regarding graft failure in patients with an MPTS <12° versus an MPTS ≥12° (15.6% vs 16.5%, respectively; *P* = .835) (Table 2).

**Table 2:** Graft Failure^*a*^

	MPTS <12° (n = 211)	MPTS ≥12° (n = 115)
No graft failure	178 (84.4)	96 (83.5)
Graft failure	33 (15.6)	19 (16.5)
*P* value	.835	.835

a Data are expressed as n (%). MPTS, medial posterior tibial slope.

Younger age (ACL graft failure: median, 22.0 years [IQR, 17.0-31.0 years]; no ACL graft failure: median, 30.0 years [IQR, 23.0-39.0 years]; *P* < .001) and male sex (ACL graft failure: 65.4% male; no ACL graft failure: 48.9% male; *P* = .034) were the only independent factors associated with graft failure (Table 3).

**Table 3:** Characteristics of Patients With Versus Without Graft Failure^*a*^

	No Graft Failure (n = 274)	Graft Failure (n = 52)	*P* Value
Age at primary reconstruction, y	30.0 (23.0-39.0)	22.0 (17.0-31.0)	**<.001**
Sex, male:female, n	134:140 (48.9% male)	34:18 (65.4% male)	**.034**
Body mass index	23.7 (21.6-25.7)	23.7 (21.3-25.6)	.671
Follow-up, mo	72.0 (50.0-105.0)	68.0 (42.0-95.0)	.236
Laterality of injury, right:left, n	127:147 (46.4% right)	26:26 (50.0% right)	.651
Time from injury to surgery, mo	8.4 (5.6-14.3)	9.4 (4.7-20.9)	.672
Graft diameter, mm	8.0 (7.5-8.5)	8.0 (7.5-8.5)	.707
MPTS, mean ± SD, deg	11.2 ± 2.8	10.6 ± 3.2	.264
IKDC score^*b*^	89.6 (45.9-100.0)	79.3 (69.8-86.2)	**<.001**
Lysholm score^*b*^	94.0 (85.0-99.0)	84.5 (75.0-90.3)	**<.001**
Tegner score^*b*^	5.0 (4.0-6.0)	4.0 (3.0-6.0)	.100
VAS for pain^*b*^	0.0 (0.0-1.0)	1.0 (0.0-2.3)	**.004**
Subjective instability score^*b*^	0.0 (0.0-1.0)	1.0 (0.8-3.0)	**<.001**

a Data are expressed as median (IQR) unless otherwise specified. Bolded *P* values indicate statistical significance. IKDC, International Knee Documentation Committee; MPTS, medial posterior tibial slope; VAS, visual analog scale.

b Data were available for 253 patients (77.6%).

Binary logistic regression showed that younger age at the time of surgery (odds ratio, 1.069 [95% CI, 1.031-1.109]) was associated with graft failure after primary isolated ACL reconstruction. Sex and MPTS did not show a statistically significant effect (Table 4).

**Table 4:** Binary Logistic Regression Analysis for Graft Failure^*a*^

	Odds Ratio (95% CI)	*P* Value
Age at primary reconstruction	1.069 (1.031-1.109)	**<.001**
Sex (female vs male)	0.603 (0.319-1.140)	.120
MPTS	1.065 (0.957-1.185)	.247

a Nagelkerke *R*^2^ = 0.11. Bolded *P* value indicates statistical significance. MPTS, medial posterior tibial slope.

### PROM

Functional outcomes, pain levels, and subjective knee stability differed significantly between patients who experienced no ACL graft failure and those who experienced ACL graft failure (Table 3). In patients without ACL graft failure, there was no correlation between the MPTS and PROM (Table 5).

**Table 5:** Correlation Analysis Between MPTS and PROM Scores in Patients Without Graft Failure^*a*^

	Spearman Correlation Coefficient (*r*_s_)	*P* Value
IKDC score	0.045	.515
Lysholm score	0.061	.371
Tegner score	0.066	.336
VAS score for pain	0.035	.608
Subjective instability score	−0.108	.115

a IKDC, International Knee Documentation Committee; MPTS, medial posterior tibial slope; PROM, patient-reported outcome measure; VAS, visual analog scale.

## Discussion

The most important finding of this study was that there was no correlation between an increased MPTS and ACL graft failure after anatomic primary isolated ACL reconstruction. Further, an increased MPTS was not associated with inferior functional outcomes in patients without ACL graft failure. Younger age at the time of primary ACL reconstruction, however, was a significant and nonmodifiable risk factor for ACL graft failure.

Although some studies also reported no association between the MPTS and ACL graft failure,^5,7,33^ several other studies have reported a significant association between the MPTS and ACL graft failure rates.^4,10,16,23,29,38^ Differences between studies may be tied to variations in (1) the definition of failure, (2) the types of patient cohorts included, (3) the surgical techniques used for ACL reconstruction, and (4) MPTS measurement methods or imaging modalities used.

Webb et al^38^ investigated the influence of the MPTS on further ACL injuries in patients after primary isolated ACL reconstruction. They reported that patients with a PTS ≥12° were 5 times more likely to suffer further ACL injuries. Previous studies,^5,7,33^ similar to our findings, also found no association between the MPTS and ACL injury recurrence or revision surgery. Beyond recurrent ipsilateral ACL injuries or revision surgery, Webb et al also included contralateral primary ACL injuries in their analysis, which may explain why the findings differed between studies. In their study, a direct comparison of the PTS between patients with ACL graft failure (9.6°± 2.3°) and those without ACL graft failure (8.5°± 2.3°) showed no significant difference, which aligns with the findings of the present study. The findings from Daehlin et al^7^ and Cooper et al^5^ should, however, be interpreted with caution, as a “nonanatomic” transtibial tunnel drilling technique was utilized in the majority of patients in the study by Daehlin et al and only patients aged ≤21 years were included in the study by Cooper et al.

Beyond the aforementioned differences, MPTS measurement methods and imaging modalities varied between studies. Lateral knee radiographs were used in most studies,^‖^ but MRI was used in several other studies.^2,4,5,14,17,19,42^ Studies that used MRI may have underestimated the MPTS by approximately 3° to 4° according to the previous literature.^15,20^ Even though an underestimation of the MPTS may not affect whether the MPTS influenced ACL graft ruptures or revision surgery, it may limit the extrapolation of study findings in which threshold values are investigated.^5,7,10,16,28,29,38,42^

Regarding the influence of other factors on ACL graft failure, young age was a significant risk factor for graft failure in the current study. This is consistent with previous findings. Zhao et al,^43^ in their systematic review and meta-analysis, reported that patients aged <25 years have 4-fold increased odds of graft failure or revision compared with patients aged >25 years. Several studies have indicated that ACL graft failure at a young age is caused by a rapid return to sports combined with high activity levels.^9,28,40,43^ Keuning et al^22^ reported an 8-fold increased risk regarding graft failure in male patients. According to several systematic reviews and meta-analyses, this difference between male and female patients is likely because of a return to high-intensity sports and a shorter time to return to play in male patients.^26,27,43^

There was no correlation between the MPTS and functional outcomes in patients without subsequent graft failure. Although PROM assess global functional outcomes of the knee, even patients with a steep MPTS showed good clinical outcomes and subjective stability. To our knowledge, the relationship between PROM and the MPTS has not been assessed previously in patients without ACL graft failure.

Some limitations of our study should be noted. First, because of the extended time frame for patient inclusion, surgical procedures were carried out by different surgeons. Nonetheless, all used the aforementioned “anatomic” technique. Second, lower leg lateral radiographs were unavailable, which led to the MPTS being measured on short lateral knee radiographs. As short lateral knee radiographs with a minimum length of 15 cm below the joint line are adequate according to the previous literature,^11^ the effect of this should not be overestimated. Third, several patients who reported ACL graft failure underwent revision ACL reconstruction, which may confound the PROM in patients with ACL graft failure.

As an increased MPTS was not associated with a higher risk of ACL graft failure in patients after anatomic primary isolated ACL reconstruction, PTS-correcting osteotomy in the context of primary ACL ruptures or graft failure after anatomic primary isolated ACL reconstruction should be reevaluated.^18,31^

## Conclusion

In patients who underwent anatomic primary isolated ACL reconstruction, the MPTS was not associated with ACL graft failure or functional outcomes. Young age at the time of primary ACL reconstruction was a significant and nonmodifiable risk factor for ACL graft failure, which should be considered in patient counseling.

## Notes

§ References 2-4, 10, 14, 16, 17, 23, 28, 29, 32, 38, 41, 42.

‖ References 3, 7, 10, 16, 23, 28, 33, 34, 37, 38, 41.
